# Hydrodynamic interactions between squirmers near walls: far-field dynamics and near-field cluster stability

**DOI:** 10.1098/rsos.230223

**Published:** 2023-06-28

**Authors:** A. Théry, C. C. Maaß, E. Lauga

**Affiliations:** ^1^ Department of Applied Mathematics and Theoretical Physics, University of Cambridge, Cambridge CB3 0WA, UK; ^2^ Physics of Fluids, University of Twente, 7500AE Enschede, The Netherlands

**Keywords:** microswimmers, biofluid mechanics, propulsion, active matter, stability, low-Reynolds-number flow

## Abstract

Confinement increases contacts between microswimmers in dilute suspensions and affects their interactions. In particular, boundaries have been shown experimentally to lead to the formation of clusters that would not occur in bulk fluids. To what extent does hydrodynamics govern these boundary-driven encounters between microswimmers? We consider theoretically the symmetric boundary-mediated encounters of model microswimmers under gravity through far-field interaction of a pair of weak squirmers, as well as the lubrication interactions occurring after contact between two or more squirmers. In the far field, the orientation of microswimmers is controlled by the wall and the squirming parameter. The presence of a second swimmer influences the orientation of the original squirmer, but for weak squirmers, most of the interaction occurs after contact. We thus analyse next the near-field reorientation of circular groups of squirmers. We show that a large number of swimmers and the presence of gravity can stabilize clusters of pullers, while the opposite is true for pushers; to be stable, clusters of pushers thus need to be governed by other interactions (e.g. phoretic). This simplified approach to the phenomenon of active clustering enables us to highlight the hydrodynamic contribution, which can be hard to isolate in experimental realizations.

## 1. Introduction

The squirmer model was first introduced by Lighthill [1] in 1952 as a simple model of a spherical Stokesian swimmer whose surface undergoes small-amplitude deformations resulting in propulsion. It was subsequently corrected and extended by Blake [2] to model a spherical microswimmer covered by a dense envelope of beating cilia; these cilia beat in a synchronized fashion, but with a phase difference which results in the propagation of a metachronal wave on the surface. The squirmer model’s idea is to replace the individual cilia with a thin, continuous layer that deforms as the wave propagates. The model was later used to describe a variety of microswimmer dynamics [3]. Biological examples include the unicellular ciliates for which the model was initially built, such as *Paramecium chlorelligerum* [4], as well as ciliate colonies including the green algae *Volvox* [5,6]. However, the interest in squirmers is not limited to the study of ciliary propulsion. Indeed, the deforming thin layer on the swimmer’s surface generates a flow that is incorporated as a slip velocity on the spherical surface. Artificial swimmers can impose identical boundary conditions to the surrounding fluid through other physical mechanisms and therefore be accurately described by the same model. For instance, phoretic Janus particles swim by inducing chemical gradients in the fluid and can be modelled as squirmers [7]. Self-propelled droplets in the bulk can also be modelled as squirmers, as the Marangoni flow on their surface generates a slip velocity [8–10].

The collective behaviour of microswimmers can differ widely from the single swimmer dynamics. Janus particles, for example, are shown experimentally to interact and form clusters [11] and are predicted to organize at large scales [12]. Self-propelled droplets can form chains of swimmers [13]. Crystal-like clusters are also observed in biological organisms, such as bacterium *Thiovolum majus* [14]. Such clustering can be mediated by the environment, including confinement [15] or single walls that reduce the dimensionality of the system [16] or bind swimmers hydrodynamically [17]. The squirmer model, sometimes refined to include varying shapes [18,19] or add a twirl to the swimmer [20] is a prime candidate to investigate such collective phenomena and interactions with the environment. Theoretical work within this framework includes studies of two-squirmer interactions [21,22] as well as the near-field stability of periodic arrays of squirmers [23]. Existing theoretical literature on squirmers echoes the significant influence of boundaries and confinement on their dynamics [24]. In particular, walls affect the speed of the swimmers [25] and can also act as temporary traps [26]. Strong confinement has been shown to stabilize hydrodynamic clusters in the near-field [27]. Extensions of this hydrodynamic model can include external cues, such as gravity and ambient flows. For example, Ishikawa *et al.* [28] computed the effective shear viscosity and normal stresses on a monolayer of bottom-heavy squirmers in a shear flow.

Many of these models aim to explain the collective dynamics seen in experiments through physical, and in particular hydrodynamic, processes alone, without taking into account biological or, in the case of artificial swimmers, chemical phenomena. Despite their significant successes, this is an essential limitation, and in particular, they cannot describe experiments where intercellular or phoretic interactions are dominant [29] and for which new models are required [30,31]. In the specific case of self-propelled droplets, variations in the surrounding chemical field are relevant for boundary interactions [32] including confinement [33], as well as long-range swimmer–swimmer interactions [29,34], collisions [35] and clustering [36,37]. Distinguishing the relative importance of hydrodynamics and chemistry in complex systems is often a very intricate task.

A notably rich system involves squirmers driven to a boundary by gravity. Experiments by Krüger *et al.* [38], Thutupalli *et al.* [39] and Hokmabad *et al.* [40] feature clusters of self-propelled oil droplets of tunable density. Bringing them to a boundary leads to the formation of stable clusters of varying sizes, from dimers and trimers to groups of several hundreds of spheres. Increased confinement leads to a destabilization of the clusters. These clusters sometimes rotate even if their shape is symmetric [40]. These experiments have inspired theoretical work combining analytical results and full hydrodynamic simulations to investigate the role of hydrodynamics as well as chemical fields. Kuhr *et al.* [41] investigated a monolayer of squirmers under gravity and observed fluctuating chains and trimers, as well as global clusters at higher packing fractions, but no intermediate hexagonal clusters. In a suspension of sedimented squirmers, dimers and trimers are observed numerically but appear to be destabilized by collisions with other swimmers [42]. Clusters are observed numerically by Rühle *et al.* [43] when the sedimented squirmers are bottom-heavy. Finally, Thutupalli *et al.* [39] consider droplets swimming vertically, and recover clusters due to pusher-pusher attraction at a boundary [44], which is modulated by the boundary condition at the wall or interface. Extensive computational works shed a light on collective dynamics, but isolating the role of each parameter and the underlying interactions can be complex and computationally time-intensive, so the range of parameters explored is often discrete.

In these studies, the squirmer parameter *β* is of particular interest; it represents the leading-order flow generated by the swimmer model, and in particular distinguishes between pullers (*β* > 0), pushers (*β* < 0) and neutral swimmers (*β* = 0). An additional distinction exists between weak and strong pullers and pushers for, respectively, low and high values of |*β*|, with a transition around unity. Many models only consider one squirmer of each category rather than a continuous variation. However, in the original experiments [38], the drops are all weak pushers, with an estimated range of squirmer parameter *β* ∈ [ −0.4, −0.2] [45], and new features appear to arise within this weak-squirmer range. Shen *et al.* [42] carried out a more complete analysis for the simpler case of a single squirmer close to a boundary, while the work of Rühle *et al.* [46] couples the simulations to analytical predictions. Some single squirmer models also include phoretic interactions [47].

The experiments of Krüger *et al.* [38], Hokmabad *et al.* [40] and related theoretical works form the inspiration for the present paper, which explores the hydrodynamic interactions of microswimmers in the presence of a single wall and under strong confinement. Our goal is to identify and isolate the purely hydrodynamic features in this system through a theoretical analysis of tractable symmetric systems. Rather than precisely reproducing a squirmer suspension’s dynamics, we hope to outline the conditions and features of the behaviours of interacting pairs and groups of swimmers. Our results are therefore meant to highlight, or rule out, hydrodynamics as the prime factor driving clustering for a set of parameters. In doing so, we aim to clarify when hydrodynamics can explain the interactions between squirmers and their prolonged clustering after they collide, and serve as the basis for further numerical and theoretical exploration of this system.

The paper is organized as follows. We first summarize and extend existing results on a single squirmer above a boundary in §2. The squirmers are driven to the wall by gravity, which induces speeds comparable to their self-propulsion. We then consider in §3 the far-field interactions of a pair of squirmers in a plane, mediated by a boundary, in two dimensions. We show that collisions between squirmers are to be expected even at low densities, regardless of the squirmer number. For pullers, these collisions occur because of hydrodynamic attraction at short and intermediate distances. For pushers, on the other hand, while hydrodynamic interactions are repulsive they act on typical timescales much longer than swimming, so collisions still take place at sufficient concentrations. However, this would not be sufficient to form sustained clusters and interactions that occur in the near-field after encounters are decisive for collective behaviour. Furthermore, experiments [38] and theory [48] have shown that clusters can be stable even when pairs of swimmers are not. Consequently, we next focus in §4 on the lubrication interactions involving two or more squirmers after they collide. We again analyse the symmetric configuration, which for such a cluster is the circular one. We explore its stability depending on the hydrodynamic properties encoded in the squirmer parameter *β*, and the presence of walls (one if gravity is strong, two for a confined system). We finally compare our analysis for a circle to some more compact cluster configurations.

## 2. A single squirmer above a rigid boundary

To isolate swimmer-boundary interactions, we first consider a single squirmer under the action of gravity and above a no-slip surface. The dynamics of single squirmers allow us to model the behaviour of squirmers in a dilute suspension before they interact in the near field. In particular, we are interested in determining the equilibrium height and orientation of a single squirmer above a wall. In this section, we outline the notation and hydrodynamic fundamentals that we will extend to multiple squirmers in the following sections. We follow here results from previous publications, in particular Rühle *et al.* [46] in the far-field and Lintuvuori *et al.* [25] in the lubrication limit.

### 2.1. The squirmer model

**Figure 1.**
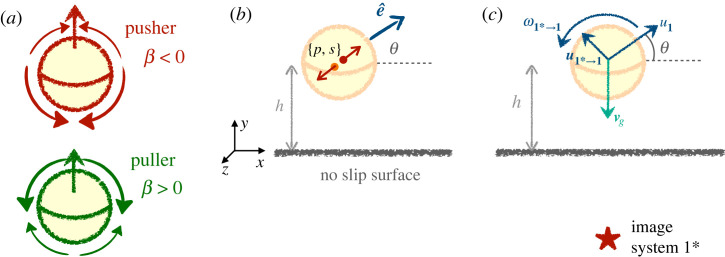
A single squirmer above a wall: (*a*) sketch of the surface flow from the two first modes around a pusher (*β* < 0) and a puller (*β* > 0). (*b*) Hydrodynamic singularities and parameters for the squirmer above a boundary. The far-field flow corresponds to a force dipole of strength *p* = −3/4*βv*_0_ *R*^2^ and a source dipole of strength *s* = *v*_0_*R*^3^/2, both oriented along the swimmer axis $\hat{e}$. (*c*) Velocity from self-propulsion ***u***_1_, gravity **v**_*g*_ and image system ***u***_1→1*_ as well as vorticity from the image system 1* ***ω***_1→1*_ acting on the squirmer.

Consider microswimmers of small typical size *L* and propulsion velocity *U*. Viscous effects dominate the fluid flow they generate since the ratio of inertial to viscous effects is small; this is quantified by the Reynolds number, *Re* = *ρ*_*f*_ *UL*/*μ*, with *ρ*_*f*_ and *μ* the density and viscosity of the fluid. The Reynolds number of self-propelled droplets with a diameter *L* ≈ 50 μm, is *Re* ≈ 10^−3^ in [38] and that of the largest *Volvox* colonies is less than 0.15 [6]. In the limit of small Reynolds numbers, the Navier–Stokes equations for the surrounding incompressible fluid flow u reduce to the Stokes equation [49],

2.1 $$\begin{array}{rl} - & \nabla p+\mu \nabla^{2}u=0\\ \mathrm{and}\;\; & \nabla \cdot u=0, \end{array}\},$$

where *p* is the dynamic pressure. We supplement this equation with appropriate boundary conditions to account for the decay of the flow at infinity and the no-slip condition on the rigid wall, namely $u{|}_{y=0}=0$ in the Cartesian coordinates system {*x*, *y*, *z*} of figure 1.

The next step is to choose the conditions imposed on the flow by the microswimmer. Here, we use the classical squirming condition [1,2], which is defined as a sphere of radius *R* with a prescribed velocity distribution on its surface. For simplicity, we only consider motility driven by a time-independent tangential slip velocity [23]. We consider squirmers with an axis of symmetry denoted by the unit orientation vector $\hat{e}$. In its general form, using spherical coordinates of polar angle $\hat{\theta }$, the velocity at the boundary is non-zero only along the polar direction ${\hat{e}}_{\hat{\theta }}$ and reads

2.2 $$u_{\hat{\theta }}{|}_{r=R}=\sin\hat{\theta }\sum_{n=1}^{\infty }\frac{2}{n(n+1)}B_{n}P_{n}^{\prime }(\cos\hat{\theta }),$$

where *P*_*n*_ is the *n*th Legendre Polynomial. All terms of this flow are characterized by the magnitude of the squirmer modes *B*_*n*_, assumed to be time-independent. We further assume that the microswimmers are force and torque-free. This yields a relation between the coefficient of the slowest decaying term, which applies a net force on the fluid, and the swimming speed in an infinite fluid *v*_0_ as *v*_0_ = 2 *B*_1_/3 [3]. Additionally, a common assumption is to retain only the first two coefficients, *B*_1_ and *B*_2_ [3,21]. The speed at a point $R{\hat{e}}_{r}$ on the swimmer surface can finally be rewritten as a function of the propulsion speed, the orientation of the swimmer $\hat{e}$, and the dimensionless ratio of the two modes *β* = *B*_2_/*B*_1_ as

2.3 $$u{|}_{r=R}=\frac{3}{2}v_{0}(1+\beta \hat{e}\cdot {\hat{e}}_{r})[(\hat{e}\cdot {\hat{e}}_{r}){\hat{e}}_{r}-\hat{e}],$$

where *β* is the squirmer parameter presented in the introduction. Because it sets the direction of the tangential flow, *β* is the main parameter that governs hydrodynamic interactions between swimmers and with boundaries. We illustrate in figure 1 the boundary conditions on the squirmer surface for pushers and pullers. This work is motivated by experiments on clustering of active droplets [38,40] which can be modelled as weak pushers, with *β* ∼ −0.1. In what follows, we will therefore focus on pullers and pushers with |*β*| < 2, and study the range of their hydrodynamic interactions.

In addition to *β*, a second parameter is needed to quantify the strength of gravity. The detailed height-dependent impact of gravity is addressed in the following sections. Here, we introduce the ratio of the gravity-induced speed *v*_*g*_ in the bulk and the self-propulsion *v*_0_. When *α* = *v*_0_/*v*_*g*_ is slightly below unity, the swimmer in the bulk is expected to be driven downwards by gravity regardless of its orientation; novel dynamics in the proximity of walls were indeed observed for single squirmers in this range of *α* both experimentally (*α* ∼ 0.8 for the active droplets in [38]) and in theory and simulations [46].

The force and torque-free conditions have been invoked so far to determine self-propulsion. In what follows, interactions with boundaries as well as with other swimmers will provide additional hydrodynamic stresses, mediated by the alteration of the external fluid flow created by both swimmers and boundaries. For a freely suspended spherical particle, we can use Faxén's law to determine exactly the velocity U and angular velocity Ω of the sphere located instantaneously at $r_{0}$ in an infinite external flow $u_{a}(r)$ [49] as

2.4 $$U=u_{a}(r_{0})+\frac{R^{2}}{6}\nabla^{2}u_{a}{|}_{r_{0}}=u_{a}(r_{0})+O(R^{2}\nabla^{2}u_{a}{|}_{r_{0}})$$

and

2.5 $$\Omega =\frac{1}{2}\nabla \times u_{a}.$$

For a swimmer at a distance *h* > 2*R* away from a surface or another swimmer, we may neglect the second (higher-order) term in the velocity equation, equation (2.4), which has a relative scale of ∼*R*^2^/*h*^2^ ≪ 1 compared to the first one. In what follows, we can use these relations to investigate both the equilibrium and dynamic orientation, height above the surface, and distance between swimmers. It will also be interesting to determine the preferred orientation of a squirmer when it is held at a fixed position with respect to other swimmers or boundaries: in such cases, only the torques exerted on the swimmer will be required to be zero.

In the next part, we focus solely on a single squirmer driven to a boundary by gravity. Sections 2.2 and 2.3 provide detailed modelling in the far-field and near-field, respectively. In the far field, we use the hydrodynamic singularity decomposition of the squirmer’s first two modes to obtain the forces and torques from hydrodynamic interactions between swimmers and with walls, expanding upon the work from Spagnolie & Lauga [50]. In the near-field, we use the lubrication torques computed by Brumley & Pedley [23] and Lintuvuori *et al*. [25].

### 2.2. Far-field hydrodynamic interaction of a single squirmer with a wall

#### 2.2.1. Velocity and angular velocity components

When the height *h* of the swimmer above a boundary satisfies *h* > 2*R*, hydrodynamic interaction may be described in the far field. The contributions to the speed and rotation of an isolated swimmer include self-propulsion, sedimentation through gravity, and hydrodynamic interactions with the wall, as illustrated in figure 1.

The contribution due to gravity generates a downward speed *v*_*g*_ in the bulk along the vertical axis, here *y*, which is modified by the boundary. Using work from the early 1900s by Lorentz [51,52] and Faxén [53], it is a classical result that the height-dependent velocity along the *y*-direction can be computed in powers of *R*/*h* [54]; we obtain to third order

2.6 $$v_{g}(h)=v_{g}\left[1-\frac{9R}{8h}+\frac{1}{2}{\left(\frac{R}{h}\right)}^{3}\right]{\hat{e}}_{y}.$$

The bulk sedimentation velocity *v*_*g*_ depends on the mismatch between swimmer density *ρ*_*s*_ and that of the surrounding fluid *ρ*_*f*_, in the form *v*_*g*_ = 2*gR*^2^(*ρ*_*s*_ − *ρ*_*f*_)/(9*μ*) for a spherical swimmer. In the following, unless otherwise mentioned, we used the value of *α* = *v*_0_/*v*_*g*_ = 20/26 ≈ 0.77 based on oil-droplet experiments of [38]. The value *α* < 1 indicates that the swimmer always sediments towards the surface and cannot completely escape the wall; furthermore, self-propulsion and gravity are of the same order of magnitude, thereby enabling floating and complex hydrodynamic interactions with the boundary and other swimmers.

As opposed to gravity, hydrodynamic interactions with the wall modify the flow of the swimmer and generate in general both a force and a torque; the requirement of free-swimming then leads to a change of both the linear and angular velocities of the swimmer. To compute their values, we write the flow field created by the squirmer in the far field as a combination of hydrodynamic singularities, namely a force dipole and a source dipole. The slowest decaying term is purely radial and decays spatially as 1/*r*^2^; it is a symmetric force dipole, or stresslet, of strength *p* = −3/4*βv*_0_*R*^2^ oriented along the instantaneous swimming direction $\hat{e}$. Because it is the flow singularity that decays the slowest, the stresslet is a distinctive feature of the flow of the vast majority of microswimmers [3]. Its sign characterizes pullers (*p* < 0) and pushers (*p* > 0), similarly to the above flow description of a squirmer type based on the sign of *β*. The second term decays as 1/*r*^3^ and corresponds to a source dipole of strength *s* = *v*_0_*R*^3^/2 also oriented along the swimmer axis $\hat{e}$. The far-field velocity created at a point r by a squirmer located at the origin can therefore be written as

2.7 $$u(r)=-\frac{\;p}{r^{2}}[1-3{\left(\hat{e}\cdot \hat{r}\right)}^{2}]\hat{r}-\frac{s}{r^{3}}[e-3(\hat{e}\cdot \hat{r})\hat{r}],$$

with r=|r| and $\hat{r}=r/r$.

Writing the flow of the squirmer through hydrodynamic singularities located at the centre of its spherical body rather than through the surface velocity immediately simplifies incorporating the no-slip boundary condition at the wall. The wall contribution is indeed mathematically equivalent to an appropriate combination of hydrodynamic singularities placed at the mirror location of each initial singularity [55]. Using the decomposition above, we thus get that the image system for the squirmer is the sum of the image systems for the force dipole and source dipole, and in turn, these mirror images create a flow at the position of the squirmer. The contributions of the hydrodynamic image of a far-field swimmer to its own speed and rotation are given in [50]. We re-derive these results in the electronic supplementary material extending them to obtain the contribution of the hydrodynamic image in the entire space, which we use in calculations involving several swimmers.

#### 2.2.2. Equilibrium states

In this section, we exploit the results on the height and orientation of a single squirmer above a boundary originally derived by Rühle *et al.* [46]. In these preliminary results, we focus on the long-time steady state of the system, as it will be used as the initial conditions for systems involving multiple squirmers in the following section §3. When equilibrium situations exist, they are characterized by a pair of stable floating height, h*, and orientation, θ*.

*Orientation.* Let us first consider the possible stable orientation of a squirmer at a fixed height *h* above a boundary, distinguishing between pushers and pullers. The reorientation rate of the squirmer is half of the vorticity from the image system, *ω*_1*→1_, and we look for fixed points in the orientation angle *θ*. Among these fixed points, the stable ones also have that ∂*ω*_1*→1_/∂*θ* < 0.

Pushers that have sedimented above a wall always have stable orientations (within the far-field approach). If *h* < 2 *R*/3|*β*|, the swimmers are oriented upwards i.e. with θ* = *π*/2. When *h* > 2 *R*/3|*β*|, the pushers become tilted with stable angle

2.8 $$\theta^{*}=\frac{\pi }{2}-\mathrm{acos}\left(\frac{-2R}{3\beta h}\right).$$

**Figure 2.**
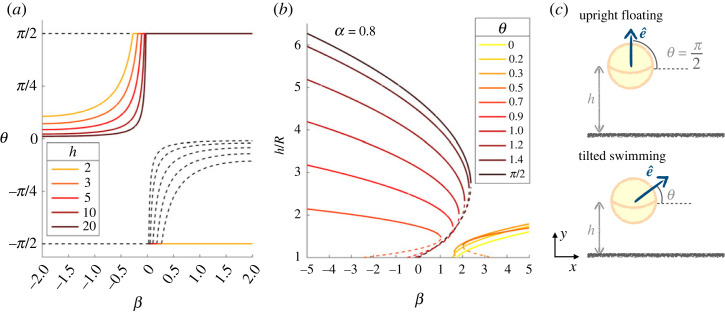
(*a*) Far-field stable equilibrium orientation of weak pushers and pullers, depending on *β* for different heights. The dashed lines represent unstable solutions. (*b*) Far-field equilibrium heights, both stable (solid line) and unstable (dashed) for different orientations. (*c*) Sketch of the two possible equilibrium states, upright floating and tilted swimming.

For pullers, on the other hand, the stable equilibrium orientation in the far field is vertical. Upright (*θ* = *π*/2) pullers are at equilibrium for any height, while downright (*θ* = −*π*/2) stable equilibria are possible if the puller is sufficiently strong *β* > 2*R*/(3*h*). We show in figure 2 the equilibrium orientations for different height *h* and squirmer parameter *β*.

*Height above the surface.* Moving on to the swimming height, the total vertical velocity of a squirmer up to terms in (*R*/*h*)^3^ is given by

2.9 $$u_{z}=v_{0}\left[-{\left(\frac{R}{h}\right)}^{3}\frac{1}{2}(\sin\theta +\alpha^{-1})+{\left(\frac{R}{h}\right)}^{2}\frac{9\beta }{32}(1-3\sin^{2}\theta )+\left(\frac{R}{h}\right)\frac{9}{8}\alpha^{-1}+\sin\theta -\alpha^{-1}\right].$$

At a fixed inclination angle *θ*, this directly yields an equation for equilibrium *h* which is found by solving a third-order polynomial. The stability of the fixed point on the other hand is assessed from the sign of the derivative. The fixed point is stable if ∂*u*_*z*_/∂*h* < 0. For the far field to be a coherent approximation, we look for swimming heights *h* > 2*R*. Examples of equilibrium heights for a set orientation are shown in figure 2*b*.

**Figure 3.**
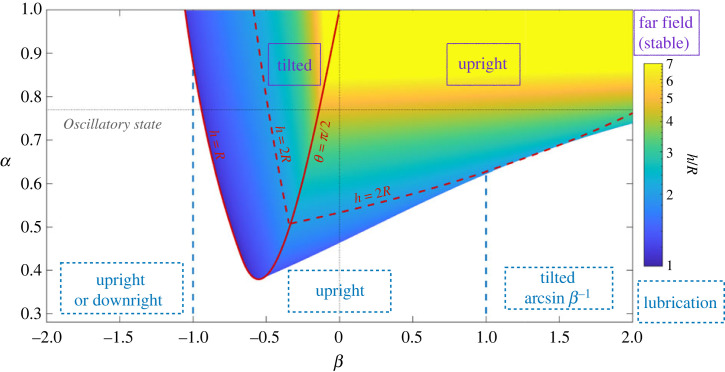
Possible equilibrium states for a single swimmer above a boundary. Far-field equilibria are in purple and solid boxes and correspond to a stable pair of orientation θ* and height *h*. Near-field equilibria are in blue and in dashed boxes and only account for the orientation. The different lubrication regimes are separated by vertical dashed blue lines, for *β* = −1 and *β* = 1. The colourmap shows the stable height of these far-field equilibria, which are stable solutions of equations (2.8), (2.9), when they exist. The solid red line delimits the tilted equilibrium region. On its right, weak pushers and pullers swim upright, with the boundary given by equation (2.10). On its left, for strong pushers, the tilted swimming corresponds to a hovering height *h* < *R*, while the upright position is unstable. As close proximity to the wall leads to upward lubrication reorientation, we expect oscillations. The dashed red line is *h* = 2*R* and indicates the validity of the far-field approximation. Dotted black lines show pusher/puller separation *β* = 0 and the value of gravity used in the rest of the paper, *α* = 0.77.

*Combined equilibria.* To determine pairs of stable equilibrium height and orientation (when they exist), we solve simultaneously equations (2.8) and (2.9) for a range of dimensionless gravity *α* and squirmer strengths *β*. The equilibrium states are plotted as a phase diagram in figure 3, together with the corresponding height *h* obtained numerically. In addition, the Matlab code used to obtain figure 3 is accessible in the repository [56], with details of the functions in electronic supplementary material.

For pushers, these far-field equilibrium stable states exist only in the weak case, *β* > −1.05. They are of two different kinds, which reflect the possible equilibrium orientations: (i) upright floating at a constant height or (ii) tilted swimming where the swimmer is inclined as in equation (2.8) and swimming above the boundary; both are sketched in figure 2c. At the transition between upright and tilted pushers, we expect to have θ* = *π*/2 and *R*/*h* = −2*β*/3. Using equation (2.9) then yields an expression for the self-propulsion to gravity ratio *α* as a function of *β*, which may be simplified to obtain the boundary in the *α* − *β* domain

2.10 $$\alpha =-4(27\beta^{3}-27\beta -16){\left(27\beta^{3}+64\right)}^{-1},$$

as plotted on figure 3. The ratio of radius to swimming height at equilibrium is found as the root of a polynomial resulting from the equation for vertical velocity, with sin *θ* = 1 in the upward state and sin *θ* = −2*R*/(3*βh*) if inclined. When increasing the relative importance of gravity (*α*^−1^) or the pusher strength (|*β*|), the swimming height decreases and reaches values *h* < *R*. The boundary is found by setting *h* = *R*, as displayed in the phase diagram.

For stronger pushers, no far-field equilibrium exists. The upright state is unstable, but the tilted one brings the squirmer down to the wall. There, near-field interactions would take over, and reorient it upright again. We therefore predict oscillations to arise in the region of strong pushers and intermediate to low gravity of figure 3. Simulations by Rühle *et al.* [46], including the impact of noise and time-dependence, indeed show that strong pushers (with *β* = −2 and *β* = −5 in their simulations) are in a bistable state and oscillate between inclined sliding at low *h* and upright floating for *θ* = *π*/2.

Weak pullers are found upward, as expected from the orientation equation, and at *h* > 2*R* for moderate gravity (0.5 < *α* < 1). With increased gravity, upward swimming no longer balances sedimentation and the squirmer sinks. In this case, the near-field interaction takes over, and depends on the precise interaction between the squirmer and the wall [25].

Overall, we therefore see that weak squirmers subject to moderate gravity exhibit two types of far-field equilibrium orientation and height: tilted swimming and upright floating, as sketched in figure 2*c*. When considering swimmer encounters in §3, we, therefore, start our analysis at these positions as initial conditions.

### 2.3. Near-field hydrodynamics of a single squirmer close to a wall

When increasing gravity, or the strength of the squirmer, the approach above predicts the sinking of individual swimmers to heights less than their radius, at which point the far-field approach breaks down. We can use a lubrication analysis to determine the orientation of these sunk swimmers when *R* < *h* < 2*R*.

We follow the work of Lintuvuori *et al.* [25], which compares simulations and theory for a squirmer close to surfaces (including the effect of repulsion). The authors show that in the near-field, lubrication controls the reorientation of the swimmers, while its velocity still depends on long-range interactions. Here, we focus only on the resulting orientation of the squirmer [46]. The near field wall-induced angular velocity was computed as [25]

2.11 $$\omega_{1^{*}\to 1,z}^{NF}=-\frac{3v_{0}}{2R}\cos\theta \;(1-\beta \sin\theta )+O\left(\ln\left(\frac{R}{\left(h-R\right)}\right)\right).$$

In this regime, the upward orientation is always a fixed point, and it is stable when *β* < 1, while stronger pullers, *β* > 1, instead have a stable equilibrium tilt with angle $\arcsin(\beta^{-1})$. A second downward stable equilibrium exists for pushers with *β* < −1, at *θ* = *π*. We added these lubrication equilibrium states in the phase diagram of figure 3.

## 3. Two-dimensional symmetric encounter of two squirmers in the far field

### 3.1. Motivation and background

We now take into account the presence of additional swimmers to gain insight into the collective dynamics of dilute suspensions. We consider the case of two swimmers in a symmetric far-field encounter. While the squirmers create three-dimensional flows, we take both their orientation vectors in the vertical (*x*–*y*) plane and symmetry therefore ensures that their orientation vector remains in the (*x*–*y*) plane. Note that this is a key simplification since interactions in the third dimension can be dominant if this planar configuration is unstable [21,43]. Our analysis will yield results on the attractive or repulsive nature of the pairwise interactions, as well as on the time evolution of swimmer orientation and therefore swimming height when in the presence of other swimmers. This modelling approach aims to complement full hydrodynamic simulations by isolating specific hydrodynamic components of the interaction of a squirmer pair above a boundary; its limitations will be addressed in the following sections.

**Figure 4.**
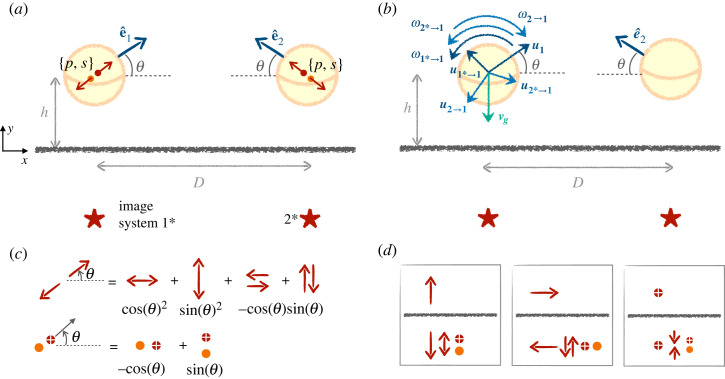
In plane encounter between two squirmers separated by a distance *D*, and with symmetric orientations, *θ*, swimming at a height *h* above a rigid wall. (*a*) Each swimmer perturbs the fluid with two hydrodynamic singularities, namely a force dipole of strength *p* and a source dipole of strength *s*, and the no-slip condition is enforced using the image system on the other side of the wall, at locations shown with stars. (*b*) Both linear and angular velocities balance on the first squirmer. It self-propels with speed $u_{1}$, and sediments at speed $v_{g}$, while being advected by the flows from its interaction with the wall, given by $u_{1^{*}\to 1}$, as well as by the second swimmer and its image system, given by $u_{2\to 1}$ and $u_{2^{*}\to 1}$, respectively. Similarly, reorientation occurs through hydrodynamic interaction with its own image system, and the other swimmer and its image, with angular velocities $\omega_{1^{*}\to 1}$, $\omega_{2\to 1}$ and $\omega_{2^{*}\to 1}$, respectively. (*c*) The hydrodynamic image systems in the far field are computed by decomposing the force dipoles and source dipoles, and (*d*) combining the image systems for parallel and perpendicular stokeslets and point sources (e.g. [55]).

In what follows, we set gravity to *α* = 0.77 to match experimental data of [38], and focus on the range of *β* where a far-field equilibrium with the wall exists for an isolated swimmer. We assume that the spherical swimmers are initially positioned at their one-swimmer equilibrium as derived in the previous section, and that, if inclined, they are facing each other so that they do not immediately separate. This configuration is shown in the left sketch of figure 4. We use the frame of reference {*x*, *y z*} (with unit vectors ${\hat{e}}_{x}$, ${\hat{e}}_{y}$ and ${\hat{e}}_{z}$). The first swimmer is oriented along ${\hat{e}}_{1}=\cos\theta \;{\hat{e}}_{x}+\sin\theta \;{\hat{e}}_{y}$, and the second along ${\hat{e}}_{2}=-\cos\theta \;{\hat{e}}_{x}+\sin\theta \;{\hat{e}}_{y}$, where *θ* is again the polar angle with respect to the horizontal (figure 4). The orientation vectors of the squirmers remain in the (*x*–*y*) plane, so their velocity is in this plane and they can rotate around the *z*-axis. The additional parameters describing the pair of squirmers are their height *h* above the surface, and the distance between the centre of the two spheres, *D*. The hypothesis of far-field interactions between the swimmers corresponds to both *h* > 2*R* and *D* > 2 *R*, as depicted in figure 4.

By symmetry, we focus only on the first spherical swimmer, which is force- and torque-free, and use Faxén's relations to find its linear and angular velocities. Contributions to the dynamics of each swimmer come from self-propulsion, the wall and the presence of the other swimmer (which creates a flow, which is itself modified by the presence of the wall). We use the same method as for the single squirmer case to quantify this new hydrodynamic contribution: (i) we decompose the far-field flow of the second swimmer into a stresslet and a source dipole, (ii) we enforce the no-slip boundary condition using the hydrodynamic image system and (iii) the combined flow from these singularities both translates and reorients the first swimmer. The detailed calculations are carried out in the electronic supplementary material and, here, we only outline their main steps.

To obtain the flow generated by the tilted Stokes dipole image, we first focus on a single-point force (stokeslet flow). Blake [2] derived its image system, which consists of an opposed stokeslet, a force dipole, and a source dipole, as sketched in figure 4*d*. The flow of a force dipole is then derived by taking two opposite stokeslets whose distance tends to zero, and the image system can be built in the same way [3]. However, care must be taken for the derivation in the case of a dipole tilted at *θ* ≠ 0. We decompose the force dipole into four stokeslets either parallel or perpendicular to the surface and add their contribution with the appropriate weights depending on the value of *θ* [3,50], as sketched in figure 4*c*. We also use a similar decomposition of the source dipole into two dipoles parallel and perpendicular to the surface, themselves computed as the derivative of the image of a point source [50,55] (figure 4).

In the end, the linear velocity of the first swimmer is controlled by self-propulsion $u_{1}$, gravity $v_{g}(h){\hat{e}}_{y}$, hydrodynamic interaction with the wall $u_{1^{*}\overset{}{\to }1}(h)$, and flow of the second swimmer $u_{2\overset{}{\to }1}(D)$ mediated by the wall $u_{2^{*}\overset{}{\to }1}(h,D)$. Similarly, the angular velocity stems from wall-induced rotation $\omega_{1^{*}\overset{}{\to }1}$ as well as from the second swimmer $\omega_{2\overset{}{\to }1}(D)$ and its hydrodynamic image $\omega_{2^{*}\overset{}{\to }1}(h,D)$. All of these components are shown schematically in figure 4.

### 3.2. Angular velocity

As we will show later, the encounter of the swimmers is controlled by their reorientation dynamics, rather than their velocity, so we study it first. To show the typical contributions to the far-field vorticity, we now compute the direct effect of swimmer 2 on 1, of the image of 1 on itself and the contribution of image 2*. As illustrated in figure 4*c*, the image systems are decomposed into components parallel and perpendicular to the walls, namely a parallel and a perpendicular source dipole for the source dipole, and four force dipoles for the force dipole. We compute the effect of the images on both swimmers. The interaction of a squirmer with the image from the other one is more intricate and is included directly in the final result of equation (3.3).

The flow in the far-field from the second swimmer $u_{2}(r)$ is the sum of the stresslet and source dipole, as given in equation (2.7). We compute its vorticity at r=(x,y,z), and evaluated it at (− *D*, 0, 0), and obtain

3.1 $$\omega_{2\overset{}{\to }1}=\left(\begin{array}{c} 0\\ 0\\ \frac{6p}{D^{3}}\sin\theta \cos\theta \end{array}\right).$$

From the calculation of the far-field flow of the squirmer image described above and detailed in electronic supplementary material, we obtain the contribution of its own hydrodynamic image to a squirmer rotation as

3.2 $$\omega_{1^{*}\overset{}{\to }1,z}=\frac{-6p}{8h^{3}}\sin\theta \cos\theta +\frac{6s}{8h^{4}}\cos\theta .$$

Summing these contributions with the one from the second hydrodynamic image computed at (−*D*, 2*h*, 0), again given in the electronic supplementary material, and taking into account the 1/2 factor of Faxén's law in equation (2.5), we finally obtain the full expression for the rate of change of the inclination angle as

3.3 $$\begin{array}{rl} \frac{d\theta }{dt} & =\frac{6phD}{R^{7}}[-20hD\sin\theta \cos\theta +2(D^{2}-6h^{2})\cos^{2}\theta +(8h^{2}-3D^{2})\sin^{2}\theta ]\\ & \;+\frac{6phD}{R^{5}}+3p\sin\theta \cos\theta \left[\frac{1}{R^{3}}+\frac{1}{D^{3}}-\frac{1}{8h^{3}}\right]\\ & \;+\frac{6s}{R^{7}}[D(D^{2}-16h^{2})\sin\theta +8h(D^{2}-h^{2})\cos\theta ]+\frac{3s}{8h^{4}}\cos\theta . \end{array}$$

### 3.3. Linear velocity

Following similar calculations, we can find the components of the linear velocity in the (*x*–*y*) plane; adding them to self-propulsion yields the total velocity of the squirmer in that two-dimensional plane. We start with the velocity induced by the hydrodynamic image of the first swimmer, obtained as

3.4 $$u_{1^{*}\to 1}=\left(\begin{array}{c} \frac{3p\sin(2\theta )}{8h^{2}}-\frac{s\cos\theta }{4h^{3}}\\ \frac{-3p(1-3\sin^{2}\theta )}{8h^{2}}-\frac{s\sin\theta }{h^{3}} \end{array}\right).$$

The second swimmer has a direct contribution at the location of the first one of

3.5 $$u_{2\to 1}=\left(\begin{array}{c} \frac{p}{D^{2}}(1-3\cos^{2}\theta )-\frac{2s}{D^{3}}\cos\theta \\ \frac{-s}{D^{3}}\sin\theta \end{array}\right),$$

while its image adds a velocity

3.6 $$u_{2^{*}\to 1}=\left(\begin{array}{c} -p\frac{\left(6h^{2}+D^{2}\right)}{2D^{4}}(3\cos(2\theta )+1)-\frac{s}{D^{4}}2(3h\sin\theta +D\cos\theta )\\ -p\frac{2h}{D^{4}}(6h\sin\theta \cos\theta -3D\cos^{2}\theta +D)+\frac{s}{D^{4}}(6h\cos\theta +D\sin\theta ) \end{array}\right).$$

Finally, *v*_*g*_(*h*) drives sedimentation along the *y*-axis as given in equation (2.6), with *α* = 0.77 in what follows (so the bulk self-propulsion is weaker than gravity and a single squirmer cannot escape the wall). The total velocity of squirmer 1 is hence the sum of these four terms, namely $u_{\to 1}=v_{g}{\hat{e}}_{z}+u_{1^{*}\to 1}+u_{2\to 1}+u_{2^{*}\to 1}$.

### 3.4. Time-evolution of orientation and height at fixed distances

Considering the far-field equilibrium configuration for the pair of spheres enables us to gain intuition on the interaction of multiple swimmers since pairwise interactions play a leading role in the dynamics of dilute suspensions. From the above results on rotational speed and velocity, we now analyse two aspects of the system: its equilibrium when the swimmers are held at a fixed distance *D*, and its dynamics during an encounter. We perform simulations and compute the positions and orientations of the two squirmers at each time step by summing the contributions to the velocities listed above in equations (2.6), (3.4), (3.5), (3.6), while the reorientation is given by equation (3.3).

**Figure 5.**
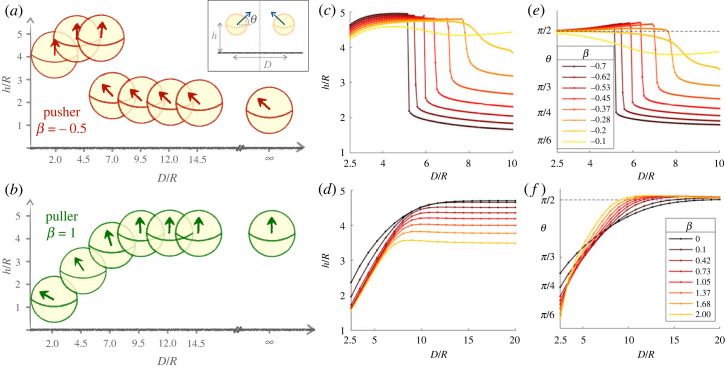
Stationary height *h* and orientation *θ* for a symmetric pair of pushers (top) and pullers (bottom) held at a fixed distance *D*, for different values of *D*/*R*, where *R* is the radius of the spherical swimmers. The squirmers are initially at the single-squirmer stationary state as in figure 3, and *θ* and *h* evolve until they reach a new coupled stationary state. The values of the squirmer parameters *β* for pushers (*c*–*e*) and pullers (*d*–*f*) are given in the inset of (*e*) and (*f*), respectively. The right swimmer is shown in (*a*,*b*), with a symmetric configuration for the left swimmer (see inset). The other panels show the final height (*c*,*d*) and orientation (*e*,*f*) of the pair of swimmers. As they get closer to each other, pushers tend to reorient upward, while the pullers tilt and sink. This transition is continuous with *D* for pullers but instead exhibits a sharp transition for pushers.

In this section, we first consider the equilibrium of two symmetric squirmers held at a distance *D* as a function of their squirmer parameter *β*. We focus on the equilibrium (computed earlier) reached when both start from their one-swimmer equilibrium (h*, θ*), for moderate gravity *α* = 0.77. Numerically, we let the orientations and heights of the squirmers evolve while keeping their separation constant until the system reaches a stationary state (*h*, *θ*). The resulting values of (*h*, *θ*) as a function of the dimensionless distance, *D*/*R*, are shown in figure 5.

The dynamics between pushers and pullers are different, so we address them separately. For pushers, and recalling the one-squirmer far-field equilibrium states, we expect to see distinct features between pushers that are initially oriented upwards and those that are hovering at an angle. Given that they are initially inclined when swimming above a boundary, the tilted-hovering swimmers always start facing each other. As we decrease the value of *D*, we see from figure 5*a* that the swimmers slowly become slightly reoriented upwards and thus swim at a higher height above the wall. Below a critical distance *D* (whose value decreases with |*β*|), the pushers undergo a sharp transition to nearly upward orientation with *θ* above *π*/2, which is associated with much higher hovering heights. At that point, the interaction between the swimmers becomes repulsive, and they are pointing slightly away from each other, as seen in figure 5*e*. For weaker pushers that were already upward on their own in the far field, the sharp lift-off transition disappears but is replaced with a dip in height and orientation at intermediate distances; this is the case for *β* = −0.1 in figure 5*c*,*e*. Note that the very long range of the interaction, which extends beyond *D* ∼ 25, is larger than the scale of the plot. We note that a pair of close pushers end up in an upward position, which was observed numerically to be an unstable dynamic fixed point for strong single pushers [46].

In contrast with pushers, puller swimmers start in the far field oriented upwards; as shown in figure 5*f* the orientation *θ* is close and slightly above *π*/2 for large values of *D*/*R*. For decreasing values of *D*, pullers begin to tilt towards one another while sinking towards the surface continuously (i.e. no sharp transition as was the case for pushers).

### 3.5. Dynamic contact between two symmetric squirmers

For both pushers and pullers, the swimmer’s orientation controls its height above the surface. When oriented upward, self-propulsion balances gravity, which lifts the swimmer from the wall. As we saw above, the primary contribution of hydrodynamic interaction is in its reorientation (as opposed to its impact on the speed). However, such reorientation is not instantaneous, and we expect the dynamics of the pair of squirmers to differ significantly from the equilibrium situations addressed above. Specifically, we expect a competition between the timescale of collision or repulsion, set by self-propulsion, and the reorientation which is governed by hydrodynamic interaction; in particular, the sharp transition observed for pushers is likely to be affected by propulsion.

We address these two effects using numerical simulations of two symmetric spherical swimmers initially positioned at a distance *D* from each other and starting from one-swimmer equilibrium. The mirror-image symmetric positions and orientations of the two swimmers evolve in time according to the previously derived velocities, in equations (3.4) to (3.6) and rotation speed in equation (3.3). To prevent overlap of the spheres at close distances, we add a repulsion speed that sets a minimal separation between them of 0.5 *R*. This repulsion should disappear if the swimmers are oriented upwards and therefore not moving in the lateral *x*-direction. We thus take a repulsion velocity in the *x*-direction proportional to the lateral speed of the swimmers, and scaling as the inverse of the distance between them, of the form $u_{r}=-v_{0}\hat{e}\cdot {\hat{e}}_{x}/(2(D-2R))$.

**Figure 6.**
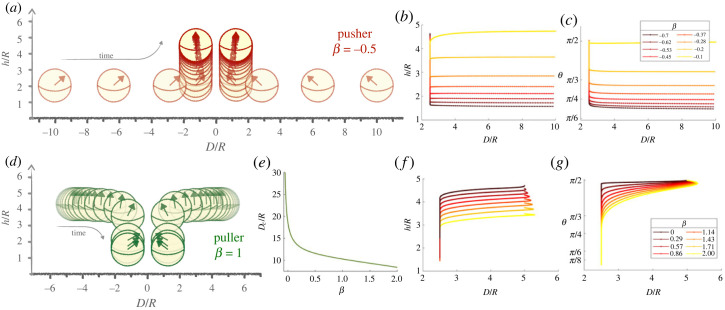
Far-field dynamics of weak pushers (*a*–*c*) and pullers (*d*–*g*). In the sketches, two symmetric pushers (*a*) (*β* = −0.5) and pullers (*d*) (*β* = 1) start at distances *D* = 10 and *D* = 5, respectively, in their single-swimmer far-field equilibria. Their time evolution is shown at dimensionless time intervals *δt* = 0.01 and *δt* = 0.2, respectively. A repulsion speed is added to ensure that the distance between the centre of the spheres *D* is above 2.5 *R* (see text). The graphs on the right show the dynamics of pushers (*b*,*c*) and pullers (*f*,*g*): evolution of height above the surface (*b*,*f*) and of orientation (*c*,*g*) for different values of the squirmer strength *β*. (*e*): minimal distance *D*_*c*_ for two upright pullers beyond which they attract each other and above which they experience hydrodynamic repulsion.

The time evolutions of the separation, height, and orientation of the squirmers are plotted in figure 6 for pushers (top) and pullers (bottom). In figure 6*a*,*d*, we show the oriented spherical swimmers at constant time intervals for a weak pusher (*β* = −0.5) and puller (*β* = 1). Once again, weak pushers and pullers are seen to have strikingly different behaviours.

Let us first consider pullers, which start floating vertically above the wall. They are set into motion by the presence of a neighbouring squirmer with an interaction that is repulsive at large distances and attractive at short distances. When placed at distances greater than a critical value *D*_*c*_, pullers repel each other and reorient slightly to move away from each other. Conversely, when placed closer than *D*_*c*_, they tilt towards each other and experience attraction. This critical distance *D*_*c*_ is set by the distance for which the equilibrium angle computed in the previous section (figure 5*f*) is less than *π*/2; the value of *D*_*c*_ is plotted in figure 6*e*, and it is shown to decrease with *β*. The existence of this critical attraction distance implies that a two-dimensional line of pullers of sufficiently high density (so that the mean distance between them falls below *D*_*c*_) would experience clustering.

Returning to the pairwise interaction, as the distance between the attractive pullers decreases, they tilt more strongly and accelerate until they meet. Because of the decrease in *θ*, the vertical contribution of self-propulsion also diminishes relative to gravity and the pullers sink (figure 6*d*). In the case of weak pullers, significant reorientation and sinking occur before contact, making far-field interactions relevant to model encounters. Note that collision occurs on timescales slower than those for pushers because pushers have initially zero horizontal velocity; this can be gleaned from the 20-fold difference in time steps used between sketches figure 6*a*,*d*. However, both reorientation and sinking continue after contact and a lubrication analysis will be necessary to understand the full collision.

In the case of pushers, our results indicate that the upward-floating type behaves initially similarly to pullers. However, contrary to pullers, pushers do not sink significantly, and their final position is upward at heights similar to the ones they started with. By contrast, pushers that are initially tilted immediately swim towards one another (figure 6*a*). This initial swimming means that by the time they collide, their orientation has scarcely changed and the lift-off predicted from the equilibrium analysis occurs after contact. The far-field formalism used here is no longer valid when the swimmers are in contact and lubrication interactions are to be included to tackle the interaction of colliding squirmers.

### 3.6. Limitations and extensions

The far-field model of a symmetric pair of squirmers that we have used so far has, by construction, two essential limitations: it lacks rotation along ${\hat{e}}_{y}$ and is not valid when the swimmers are close to one another. By choosing the initial position and orientation of the swimmers in the (*x*–*y*) plane, we confine the system to two dimensions by symmetry. However, small deviations from this initial configuration may lead to rotation along the *y*-axis. Such reorientation in the third dimension could lead the two squirmers to avoid collision [21] or show a more complicated interaction [57,58]. Nevertheless, since the reorientation of weak pushers is slow with respect to their self-propulsion and weak pullers experience short-range attraction, we expect that collisions would still occur.

A key question is then whether a collided pair of squirmers stay together dynamically. Experiments by Krüger *et al.* [38] have shown that when two self-propelled oil droplets encounter, they collide and lift off from the boundary. These droplets being weak pushers, their behaviour is in good agreement with our far-field hydrodynamic prediction shown in figure 6*a*. After a collision in the experiments, however, the droplets can float as a dimer [40] or separate and go back to isolated swimming [38]. The break-up cannot be accounted for with our current symmetric far-field model. The far-field approach taken so far cannot accurately describe the dynamics of squirmers that are close to one another. We move on to a lubrication analysis in the next section.

## 4. Near-field orientation stability of a circle of squirmers

### 4.1. Hydrodynamic interactions in a circle of squirmers

The previous far-field analysis of a pair of symmetric squirmers shows that hydrodynamic interactions with both the wall and other squirmers primarily impact their reorientation. Furthermore, as this rotation is not instantaneous but occurs at a rate slower than self-propulsion, it is expected to happen mostly in the near-field limit. We therefore extend our analysis to the case of near-field interactions between squirmers and with surrounding walls. Since we know from the literature that the collision between two swimmers can be unstable [21,38], our near-field approach needs to be three-dimensional. Furthermore, even with unstable pairs, increasing the number of squirmers involved in a collision could lead to the formation of stable clusters of several squirmers in contact. Experimentally, active droplets that are unstable in pairs can form clusters when a higher number of swimmers are included [38]. Similarly, in simulations, large numbers of swimmers can lead to cluster formation [41]. When including the third dimension and possible instabilities in groups of swimmers, the number of swimmers involved is thus likely to play a critical role.

In this section, we thus study reorientation in groups of two or more squirmers, with a particular focus on the influence of the number of swimmers in the cluster. Since the timescale for the collision of weak squirmers is much smaller than the relevant timescale for hydrodynamic reorientation, we will assume that the collision has already occurred and look at the stability of the resulting cluster. We focus on the simplest geometrical configuration where all swimmers are distributed along a circle. Since we saw that speed is primarily dominated by self-propulsion, gravity, and, when relevant, steric interactions while reorientation is driven by hydrodynamic interactions, we fix the position of the centre of the squirmers on the circle and examine their preferred orientation. These simplifications allow us to gain analytical insight and to obtain the orientation stability of the circle clusters, revealing how the number of swimmers *N* and their strength *β* control the stability of squirmer aggregates. The results for all the configurations we study are finally summarized in a table.

**Table 1.** Summary of the orientation stability of circle of squirmers in the lubrication regime for pushers and pullers, in two and three dimensions. Red backgrounds indicate instability, while green ones represent conditionally stable systems. In two dimensions (top row), the stability condition is given analytically. Importantly, two-dimensional stability is a necessary condition for three-dimensional stability. In three dimensions, we only include configurations that can be stable in two dimensions, and we distinguish between the different levels of confinement of the system (in the bulk, above one wall and between two walls). In each case, we state the nature of the instability (polar versus azimuthal) and the relevant stability conditions. The second line shows how the parameters should be varied to stabilize the circle.

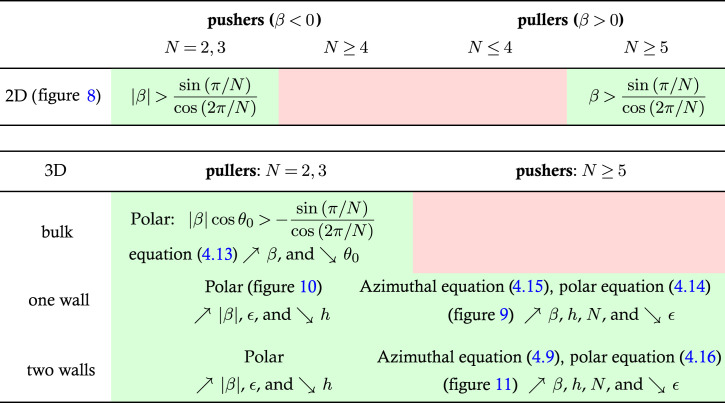

### 4.2. Near-field hydrodynamic torques

The near-field hydrodynamic interactions between two spherical squirmers come in the form of pairwise interactions between neighbouring systems that are sufficiently close to each other: swimmer–swimmer and swimmer-wall. Ishikawa *et al.* [21] and Brumley & Pedley [23] studied analytically the near-field hydrodynamic interactions between two spherical squirmers. These studies considered two adjacent spherical bodies separated by a dimensionless distance ϵ and solved the Stokes equations for a squirmer near a no-slip sphere up to the second order in ϵ . Exploiting linearity allows them to obtain the full solution to the problem and the values of the torques on each squirmer. Here, we use their results and simplify them to focus on our two cases of interest: two identical spherical swimmers and squirmer-wall interactions.

Using this past work, we can now give the hydrodynamic torques exerted on a pair of squirmers, denoted (1) and (2), and on a squirmer close to a wall [21,23]. We denote by ϵ the dimensionless ratio between the sphere–sphere or sphere-wall distance and the radius *R*.

**Figure 7.**
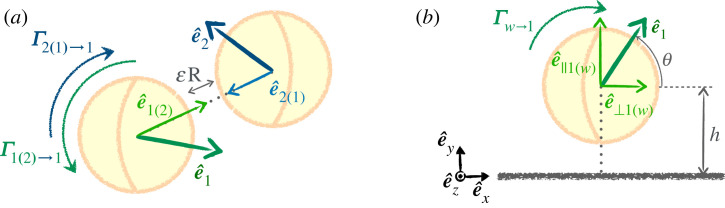
Notations the lubrication interaction between a squirmer and (*a*) another squirmer and (*b*) with a wall. Note that the orientations of the squirmers, showing using the unit vectors ${\hat{e}}_{1}$ and ${\hat{e}}_{2}$, can be out of plane.

Considering first the situation of swimmer–swimmer interactions, we see that two torques are exerted on swimmer (1) in the presence of (2). The first one is its own squirming flow that is affected by the presence of a rigid sphere at (2), which we denote $\Gamma_{1(2)\to 1}$. The second one stems from the modification of the flow of (2) due to the presence of the sphere (1) and is given by $\Gamma_{2(1)\to 1}$. Both squirmers and torques are shown in figure 7. We will use the same notation in what follows, with $\Gamma_{\alpha ,i(j)\to k}$ denoting the torque on (*k*) created by the flow of (*i*) in the presence of a rigid sphere at the location (*j*), projected on the *α*-axis, with *α* = *x*, *y* or *z*.

Writing these torques as a function of the unit orientation vectors ${\hat{e}}_{1}$ and ${\hat{e}}_{2}$, the unit oriented vectors joining the two squirmer centres ${\hat{e}}_{1(2)}=(x_{2}-x_{1})/\|x_{2}-x_{1}\|=-{\hat{e}}_{2(1)}$ and their scalar product, $e_{i(j)}={\hat{e}}_{i}\cdot {\hat{e}}_{i(j)}$ for *i* = 1, 2 (figure 7).

4.1 $$\Gamma_{1(2)\to 1}=\frac{12}{5}\pi \mu R^{2}v_{0}(1+\beta_{1}e_{1(2)})\;(-\ln\epsilon ){\hat{e}}_{1(2)}\times {\hat{e}}_{1}$$

and

4.2 $$\Gamma_{2(1)\to 1}=\frac{3}{5}\pi \mu R^{2}v_{0}(1+\beta_{2}e_{2(1)})(-\ln\epsilon )\;{\hat{e}}_{2(1)}\times {\hat{e}}_{2}.$$

In what follows, we assume that all squirmers are identical and are thus characterized by a single squirming parameter *β*. In that case, we note the relation between the torques due to the flow from (*i*) in the presence of (*j*) on each squirmer

4.3 $$\Gamma_{i(j)\to j}=\frac{1}{4}\Gamma_{i(j)\to i}.$$

The strongest effect on a squirmer in the presence of another identical swimmer is therefore (by a factor of 4) the modification of its own flow due to the presence of a rigid sphere as opposed to the flow from the activity of the second squirmer.

The effect of a wall can be similarly inferred by replacing one of the spheres with one of infinite diameter [23] (see also [25]), leading to

4.4 $$\begin{array}{rl} \Gamma_{w\to 1} & =\frac{24}{5}\pi \mu R^{2}v_{0}e_{⊥1(w)}(1+\beta e_{\left\|1(w\right)})(-\ln\epsilon )\;{\hat{e}}_{\left\|1(w\right)}\times {\hat{e}}_{1}\\ & =\frac{24}{5}\pi \mu R^{2}v_{0}(-\ln\epsilon )\cos\theta \;(1-\beta \sin\theta )\;{\hat{e}}_{z}. \end{array}$$

with vectors defined in figure 7.

We note that these equations allow us to obtain the preferred orientation for a pair of squirmers as a function of *β*. Weak (|*β*| < 1) or neutral squirmers tend to align facing opposite directions (preferred configuration: ←→); strong pushers tend to align regardless of their respective orientation (←← or →← or ←→); finally, strong pullers preferably reorient parallel to one another (↑↑ or ↑↓). These pairwise configurations provide intuition on possible configurations for *N* swimmers and the transitions between such configurations as a function of *β*.

### 4.3. Orientation stability of a circle cluster in the (*x*–*z*) plane

#### 4.3.1. Reorientation and torques

The torque exerted on a given swimmer is the sum of the pairwise interactions with all its nearest neighbours, so we can directly use the results from the previous section involving two squirmers to address the stability of *N* swimmers. The simplest configuration is a symmetric one with all swimmers placed on a circle. This is the simplest relevant geometry for the collision of more than two squirmers, or for one colliding with a small existing cluster. Its regularity allows us to derive analytical results on stable configurations, which could be extended numerically to systems without such symmetry.

**Figure 8.**
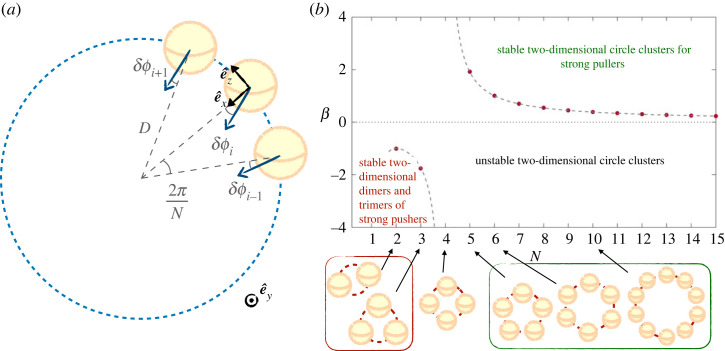
Two-dimensional stability for circle clusters of squirmers in a plane. (*a*) Notations for the angles *δϕ*_*j*_ and the local frame of reference for swimmer (*i*); (*b*) phase space of the possible stable orientations for a circle of *N* identical squirmers of strength *β*. For pushers, only dimers (*N* = 2) and trimers (*N* = 3) are stable, while for pullers, increasing the *N* stabilizes the system and stable circles can exist only for *N* ≥ 5. The points show numerical results while the dashed lines are equation (4.9).

We first consider a 2D planar system where the squirmers can reorient in the (*x*–*z*) horizontal plane, illustrated in figure 8*a*. We consider a circle of diameter *D* consisting of *N* identical squirmers, all initially pointing inwards and radially. We fix the position of the centres of the squirmers, but their orientation vectors ${\hat{e}}^{\left(i\right)}$ are free to rotate around the *y*-axis. We denote by *δϕ*_*i*_ (*i* ∈ [1, *N*]) the angle between the radial inward orientation and the orientation of the swimmers (figure 8*a*). The distance between neighbouring swimmers is Rϵ=2[Dsin⁡(π/N)−R]. The torque exerted along the *y*-axis on swimmer *i* has four components: two arise from its own flow in the presence of the neighbouring spheres *i* − 1, *i* + 1, and two from the flow created by these neighbours. Since only the pairwise lubrication interactions are relevant, each of these components can be derived from equations (4.1), (4.2), and added. The full three-dimensional expressions for the torques are given in the electronic supplementary material [61], and we use here their restrictions to two dimensions.

Let us first focus on the interaction between squirmers *i* and *i* + 1. We express everything in the local frame of reference of *i*, with ${\hat{e}}_{x}$ chosen radially oriented inward, as in figure 8*a*. From our choices of angles, we have ${\hat{e}}^{\left(i\right)}=[\cos(\delta \phi_{i}),0,-\sin(\delta \phi_{i})]$, and ${\hat{e}}_{\left\|i(j\right)}=[\sin(\pi /N),0,\cos(\pi /N)]$, so equation (4.1) leads to the torque

4.5 $$\Gamma_{y,i(i+1)\to i}=\frac{-12}{5}\pi \mu R^{2}v_{0}(-\ln\epsilon )\cos\left(\frac{\pi }{N}+\delta \phi_{i}\right)\left(1+\beta \sin\left(\frac{\pi }{N}+\delta \phi_{i}\right)\right).$$

The other three components can be deduced using appropriate symmetries and switching indices between swimmers. Except for the orientation of *δϕ*_*j*_, the three-swimmers system is symmetric with respect to the (*x*–*y*) plane. Since Γ is the cross product of two vectors, it is a pseudo vector, so the *y* component is reversed under symmetry. Switching signs and *δϕ* → −*δϕ*, we obtain

4.6 $$\Gamma_{y,i(i-1)\to i}=\frac{12}{5}\pi \mu R^{2}v_{0}(-\ln\epsilon )\cos\left(\frac{\pi }{N}-\delta \phi_{i}\right)\left(1+\beta \sin\left(\frac{\pi }{N}-\delta \phi_{i}\right)\right).$$

Similarly, we can use equation (4.3) which states that $\Gamma_{\;j\;(j-1)\to j-1}=(1/4)\Gamma_{\;j\;(j-1)\to j}$ to obtain $\Gamma_{i\;(i-1)\to i-1}$. We then switch indices and add the four individual torques to obtain the full torque exerted on the squirmer *i* along the *y*-axis as

4.7 $$\begin{array}{rl} \Gamma_{y,\to i}=\frac{12}{5}\pi \mu R^{2}v_{0}(-\ln\epsilon ) & \{[\cos\left(\frac{\pi }{N}-\delta \phi_{i}\right)\left(1+\beta \sin\left(\frac{\pi }{N}-\delta \phi_{i}\right)\right)\\ & -\cos\left(\frac{\pi }{N}+\delta \phi_{i}\right)\left(1+\beta \sin\left(\frac{\pi }{N}+\delta \phi_{i}\right)\right)]\\ & +\frac{1}{4}[\cos\left(\frac{\pi }{N}-\delta \phi_{i-1}\right)\left(1+\beta \sin\left(\frac{\pi }{N}-\delta \phi_{i-1}\right)\right)\\ & -\cos\left(\frac{\pi }{N}+\delta \phi_{i+1}\right)\left(1+\beta \sin\left(\frac{\pi }{N}+\delta \phi_{i+1}\right)\right)]\}. \end{array}$$

#### 4.3.2. Stability of the two-dimensional circle

We run numerical simulations to investigate the stability in the orientation of the circle cluster. In these, the centres of the *N* swimmers are regularly positioned on a circle of radius *R* = 1, with their orientation vectors initially pointing inwards (i.e. towards the origin). The vectors then rotate due to the hydrodynamic torques computed from equation (4.7), as well as some rotational white noise. We use a forward time stepping for the time evolution of the orientation vectors, with time step d*t* = 0.01. The noise ensures that the system does not get stuck in an unstable configuration. It induces a rotation with a direction normal to the plane of the circle for planar simulations and uniformly distributed on the unit sphere for full three-dimensional ones. We take its norm to be normally distributed with a standard deviation *η* small relative to the hydrodynamic torques, but sufficient to accelerate our simulations for very weakly unstable systems. Quantitatively, we take the noise strength to be *η* = 0.05*v*_0_ for the simulations to be efficient and check that decreasing it does not affect the resulting numerical stability range. The corresponding Matlab code can be found in the online repository [56], with details in the electronic supplementary material.

A circle of *N* squirmers of strength *β* is deemed to be stable if, in the dynamical simulations, all swimmers keep on pointing inward, so that at all times, we have $\delta \phi_{i}<\pi /2-\pi /N\;(\forall i)$. The resulting stability limit is plotted in figure 8. While circles of weak pushers are always unstable, for sufficiently stronger ones, dimers and trimers (i.e. circles of two and three squirmers respectively) are seen to be stable. This was observed numerically by Kuhr *et al.* [41] in a monolayer of sedimented squirmers, with transient dimers and trimers seen for *β* = −2, before being broken by noise or collisions with other squirmers. For pullers, on the other hand, small clusters with *N* = 2 − 5 are always unstable. For *N* ≥ 5, increasing either the number of pullers or the strength of the squirmers *β* leads to stable clusters, as shown in figure 8. Squares of squirmers (i.e. *N* = 4) are always unstable regardless of *β*, a result that can be inferred from the preferred respective orientations (aligned or parallel) of pairs of squirmers.

We can interpret these computational results theoretically by analytically determining the stability limit of the configuration where all swimmers point inwards. Different instability modes could develop to break the circle. For example, all swimmers could turn in the same direction; then, if we started with $\delta \phi_{j}=\tau \;(\forall j)$, with *τ* small, in equation (4.7), this mode would be unstable if that initial perturbation increases. Another perturbation could be of the form *δϕ*_*j*_ = ( − 1)^*j*^*τ*, where the turning direction alternates between neighbours. The simplest perturbation would be that just one swimmer starts turning, *δϕ*_*i*_ = *τ*, and instead of being reoriented towards the radial direction, it keeps rotating away, possibly bringing along its neighbours. For the system to be stable, it needs to be stable for all these perturbations, and the limit for stability is of course the limit for the most unstable mode.

A standard analysis would allow us to determine the stability range of all possible modes. Alternatively, we can focus on the modes that are predicted from our numerical simulations to be the most unstable ones. Taking only one swimmer to start turning *δϕ*_*i*_ = *τ*, while keeping *δϕ*_*i*−1,*i*+1_ = 0, turns out to be the most unstable configuration in all of our simulations. Analytically, we, therefore, focus only on this perturbation, compute the parameters for which it is unstable, and check that we obtain the right stability condition by comparing our analytical prediction with the simulations. Rearranging equation (4.7) with *δϕ*_*i*_ = *τ* > 0 and *δϕ*_*i*−1,*i*+1_ = 0 gives the torque on squirmer *i* as

4.8 $$\Gamma_{y,\to i}=\frac{24}{5}\pi \mu R^{2}v_{0}(-\ln\epsilon )\sin\tau \left[\sin\left(\frac{\pi }{N}\right)-\beta \cos\tau \cos\left(\frac{2\pi }{N}\right)\right].$$

If $\Gamma_{y\to i}$ is negative, the perturbed swimmer reorients radially and the perturbation decreases. Conversely, if it is positive, the initial angular perturbation increases and the circle configuration is unstable. This change in sign occurs when

4.9 $$|\beta |>\left|\frac{\sin(\pi /N)}{\cos(2\pi /N)}\right|.$$

We plot this analytical limit in figure 8 as dashed lines along with the numerical results. The theoretical prediction for both pushers and pullers is in excellent agreement with the computations, we check that this is indeed the stability limit for the orientation of circles of squirmers. Note that since our computations only required swimmers to remain in some inward window to be termed stable but that the stability criterion requires swimmers to point exactly radially inward for all times, we, therefore, see that when circles are stable all swimmers continuously point towards the centre of the circle for all times.

In this section, we considered stability in two dimensions. Tilting in the third dimension can weaken the instability, but not cancel it. Besides, adding confining walls parallel to the circle would induce new hydrodynamic torques on the swimmers, given by equation (4.4), but these would be oriented along $\pm {\hat{e}}_{y}\times {\hat{e}}^{\left(i\right)}$, and would not modify these stability results in the horizontal plane. Two-dimensional stability is therefore a necessary condition for three-dimensional stability both in the bulk and in the vicinity of walls. Accordingly, for the pushers in the experiments of Krüger *et al.* [38], circle configurations of four or more squirmers are always unstable. The strength of the instability is lesser for weaker pullers, such as the oil droplets in the experiments, but a mechanism distinct from hydrodynamic reorientation is nonetheless required to stabilize the clusters. In the first part of this article, we showed that the lift-off and possible break-up seen for two colliding squirmers could be explained hydrodynamically. By contrast, stable clusters seen experimentally when more droplets are brought together cannot be explained by hydrodynamic interactions.

### 4.4. Orientation stability of circle clusters in three dimensions

**Figure 9.**
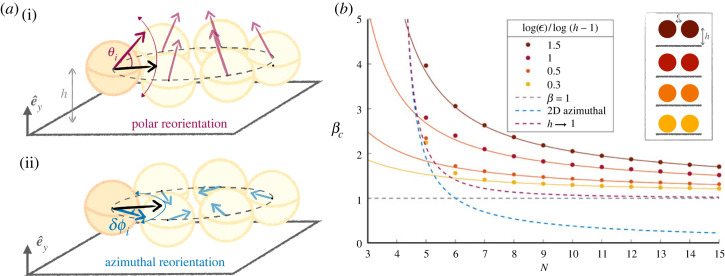
(*a*) Schematic polar (i) and azimuthal (ii) instability modes in a circle of squirmers. The reorientation can stop with squirmers still pointing inward (as shown) or continue to either *θ* > *π*/2 or *δϕ* > *π*/2 − *π*/*N* and break the circle. (*b*) Stability of a circle of pullers above a wall: critical squirmer parameter *β*_*c*_ for different spacing between the squirmers ϵ and hovering heights *h*. For each ratio ln⁡ϵ/ln⁡(h−1), the symbols show the value of *β* above which the circle is stable; insets display an example configuration. The solid lines show the critical beta value for polar stability in *θ* computed numerically from equation (4.14). As the squirmer approaches the wall, these dotted lines tend to *β* = 1 for the polar stability of a single squirmer above a wall shown in purple. The blue dashed line is the two-dimensional limit for azimuthal stability, from equation (4.9). Finally, the grey dashed line represents the global stability for a circle touching the wall at *h* → 1, as given in equation (4.15). It, therefore, lies above the two previous limit cases, but below the dots from simulations as proximity to the wall stabilizes the circle of pullers.

We now consider how allowing reorientation in the third dimension could impact the stability results. Once again, the location of the centre of each sphere remains fixed on the circle but their orientation vector can now point to any spatial direction. Is the two-dimensional planar limit for hydrodynamic stability reached in systems of three-dimensional rotating swimmers, or do new instabilities appear? The additional vertical rotation is characterized by the angle *θ* and can occur on top of the previous (*x*–*y*) rotation of *δϕ*. We choose the orientation *θ* relative to the (*x*–*z*) horizontal plane so that *θ*_*i*_ ∈ [− *π*, *π*], i.e. *θ* is the elevation angle, while *δϕ*_*i*_ ∈ [− *π*, *π*] measures the azimuthal deviation from the radial orientation as above. Variations of *θ* and $\delta_{\phi }$ are depicted in figure 9. Clusters could therefore now be broken through two instabilities, modes in the (*x*–*y*) plane and those out of the plane. We term azimuthal modes those in the plane for which |*θ*_*i*_| remains strictly below *π*/2 while |*δϕ*| increases beyond *π*/2 − *π*/*N*; in contrast for modes out of the plane, which we term polar modes, an unstable swimmer would cross the vertical orientation |*θ*| = *π*/2 before being directed outward at |*θ*| > *π*/2. Examples of these azimuthal and polar perturbations are shown in figure 9*a*.

We perform numerical simulations where we let the orientation of squirmers aligned in a circle evolve with time adding a small background white noise as above. We then regard a circle configuration as stable if all swimmers in it are still oriented inward, i.e. *δϕ*_*i*_ < *π*/2 and $\theta_{i}<\pi /2\;(\forall i)$. We then compare the numerical results with an analytical stability limit for simple configurations, allowing one reorientation at a time (either polar or azimuthal).

#### 4.4.1. Pullers

We first focus on the case of pullers. We find that all puller circles eventually come apart through the new polar instability, regardless of their two-dimensional behaviour (stable versus unstable). They are systematically rotated upwards to increasing values of |*θ*|, reaching *θ* > *π*/2 and then continuing to rotate, thus breaking the circle. For the circles that were already unstable in two dimensions, the azimuthal instability always occurs on larger time scales, so the breaking of the clusters is dominated by the polar mode. We can recover this result by examining the effect of a polar perturbation in the expressions of the pair torques of equations (4.1), (4.2). We derive in the electronic supplementary material the full lubrication torque exerted by one swimmer *i* in a circle of *N* squirmers, a result that can then be used to derive the simple approach below, and extend them to a more general case where azimuthal and polar reorientations are coupled.

We consider analytically the polar instability on its own (i.e. uncoupled to azimuthal rotation). We thus assume that all swimmers remain oriented radially at $\delta \phi_{j}=0\;(\forall j)$, but that they can be tilted by *θ*_*j*_. Let us examine how the *θ*_*i*_, the polar orientation of the *i*th swimmer, is modified by computing the torques along the *z*-axis in its own frame of reference {*x*, *y*, *z*}. First, the squirmer is reoriented by its own flow in the presence of its neighbours with viscous torque

4.10 $$\Gamma_{z,i(i\pm 1)\to i}=\frac{12}{5}\pi \mu R^{2}v_{0}(-\ln\epsilon )\left[1+\beta \cos\theta_{i}\sin\left(\frac{\pi }{N}\right)\right]\sin\theta_{i}\sin\left(\frac{\pi }{N}\right).$$

Since Γ is the component of a pseudovector, we expect by symmetry that the *z*-components from *i* − 1 and *i* + 1 to have identical contributions to *i*. For pullers, we have *β* > 0 so these torques are positive when *θ*_*i*_ < *π*/2 and thus tend to increase any tilting.

Considering the effect of the flow from the neighbouring squirmers, we find

4.11 $$\Gamma_{z,i\pm 1(i)\to i}=\frac{3}{5}\pi \mu R^{2}v_{0}(-\ln\epsilon )\left[1+\beta \cos\theta_{i\pm 1}\sin\left(\frac{\pi }{N}\right)\right]\sin(\theta_{i\pm 1})\sin\left(\frac{\pi }{N}\right).$$

For pullers pointing inward (*θ*_*j*_ < *π*/2), the torque along ${\hat{e}}_{z}$ always has the sign of *θ*_*i*±1_.

Using equation (4.3), these expressions also give us the torques on (*i* ± 1), up to a constant factor of 1/4. Therefore, if the *i*th puller is slightly tilted at a non-zero angle *θ*_*i*_ either positive or negative, the perturbation is going to increase (equation (4.10)). Simultaneously, it propagates to the neighbouring swimmers (equation (4.11)), which further reinforces the instability, which is pictured in figure 9*a*.

Puller circles in the bulk always undergo polar reorientation, and the polar mode developing is the one in which all swimmers reorient together at the same *θ*_*j*_. The stable polar angle is

4.12 $$\theta_{j}=\left\{ \begin{array}{cc} \arccos\left(-{\left[\beta \sin\left(\frac{\pi }{N}\right)\right]}^{-1}\right)\; & \text{ if }\beta >\sin\left(d\frac{\pi }{N}\right),\;\\ \pi \; & \text{ otherwise}.\; \end{array}\right.$$

In all cases, a circle of pullers (*β* > 0) under purely polar reorientation has an outward equilibrium configuration and the squirmers tend to swim away from the cluster, which is always unstable. Additional azimuthal perturbation can develop in a puller circle, notably for systems that were unstable in two dimensions, but we find that they grow slower than the polar one. Besides, the final equilibrium configuration always has *δϕ*_*j*_ = 0. In summary, polar reorientation is the dominant instability for pullers in the bulk, and the inward circle is always unstable.

#### 4.4.2. Pushers

By contrast, pusher dimers and trimers remain stable for the same values of *β* as in two dimensions. This is straightforward for dimers, as in the bulk the pair is axially symmetric and has no preferred direction. For trimers, a polar stability analysis can be carried out, and we see from equation (4.10) that *θ* = 0 is unstable only for $|\beta |<2/\sqrt{3}$, so for trimers already unstable in the azimuthal direction. Therefore, if initially oriented in the (*x*–*z*) plane, pusher dimers and trimers are stable as in two dimensions (equation (4.9)). If we include one or more squirmers initially inclined with non-zero values of *θ*, or if a non-zero *θ* is imposed externally, the torques in equation (4.7) are modified such that *β* becomes $\beta \cos\theta_{j}\;(j=i-1,\;i,\;i+1)$. If all the swimmers are inclined with the same *θ*, the limit for stability for two or three pushers becomes

4.13 $$|\beta |\cos\theta >-\frac{\sin(\pi /N)}{\cos(2\pi /N)}.$$

### 4.5. Circle of squirmers above a boundary

In dilute suspensions, the accumulation of swimmers at a boundary increases their local concentration and thus the number of collisions. For our squirmers under gravity, collisions and clustering are likely to occur close to a wall. We saw, for example, that pullers in the far field tend to sink when approaching other swimmers. Under strong gravity, a single squirmer will experience lubrication interactions with the wall, as seen in the one squirmer case in the first section. Our analysis of a single squirmer over a boundary highlighted the influence of the wall on the *θ*-orientation of a squirmer under gravity. We therefore expect the presence of the wall to affect cluster stability, by setting the height of the swimmers and modifying their polar tilting and we now include it in this near-field stability analysis.

We focus on a circle of swimmers of diameter *D* with fixed positions located at a height *h* < 2*R* above a wall, with all hydrodynamic interactions being in the lubrication limit. By symmetry, the wall does not create any additional torque in the azimuthal *y*-direction. However, as above, the projection *e*_*i*(*j*)_ of the unit vector ${\hat{e}}_{i}$ is modified when a swimmer is tilted vertically, and *β* becomes $\beta \;\cos\theta_{j}\;(j=i-1,\;i,\;i+1)$ for each component of the *y*-torque in equation (4.7). In addition, the polar reorientation now includes a component set by lubrication interactions with the wall, which was computed in equation (4.4).

We run simulations of circles of pullers and pushers, varying their strength *β*, the number *N* in the circle, the dimensionless spacing ϵ and the proximity to the wall *h*. We let the clusters evolve with time according to equations (4.1), (4.2), (4.4), with noise, starting from the configuration where the swimmers are all radially inwards and horizontal, *δϕ*_*i*_ = 0 and $\theta_{i}=0\;(\forall i)$. As above, clusters are stable if they still point inwards (*δϕ*_*i*_ < *π*/2 − *π*/*N*) at large times.

#### 4.5.1. Pullers

We first consider the case of puller circles, both numerically and analytically; recall that in the bulk, the circle in two dimensions is stable for more than five squirmers but it breaks down in three dimensions by a polar (*θ*) instability. We find that, near a wall and for *β* sufficiently high, the cluster is stabilized by lubrication interaction with the wall if it is sufficiently close to it. The dots in figure 9*b* represent the limit for numerical stability (i.e. we obtained numerically an instability for *β* above those values). The different colours correspond to different choices of swimmer separation ϵ and heights *h*, with examples shown in the inset.

Can we predict these numerical results for pullers theoretically? The first necessary condition for stability is for the wall to balance the *θ*-instability that occurs in the bulk. A stable θ* < *π*/2 for which *z*-torques in a circle cancel exists for sufficiently small ratios ln⁡ϵ/ln⁡(h−1) (figure 9). For the configuration where the squirmers all point radially inward, it corresponds to the equation

4.14 $$\frac{3}{4}\frac{\ln\epsilon }{\ln(h-1)}\sin\left(\frac{\pi }{N}\right)\tan\theta^{*}\left[1+\beta \cos\theta^{*}\sin\left(\frac{\pi }{N}\right)\right]=\beta \sin\theta^{*}-1.$$

This condition, solved numerically, is plotted for different heights as coloured lines in figure 9*b*. The polar instability dominates the circle break-up for large *N*. As the circle sediments and approaches contact with the boundary, the inclination of the swimmers *θ* is set by the wall only and therefore reaches the angle predicted for a single squirmer above a wall, $\arccos(\beta^{-1})$. In this limit, the polar instability does not break the circle of pullers with *β* > 1, as depicted in figure 9*b*.

In addition to polar stability, the azimuthal condition for azimuthal stability of a circle of pullers in the (*x*–*y*) plane given in equation (4.13) remains valid. The limit for *θ* → 0 of equation (4.9) is shown as the dashed blue line in figure 9. At moderate values of *N*, however, the azimuthal instability is favoured by the polar tilt of the squirmers. We can thus obtain a better estimate of the onset of instability by considering the azimuthal instability when *θ* is set by the wall as *h* → 1, which is given by

4.15 $$\sqrt{\beta^{2}-1}=\frac{\sin(\pi /N)}{\cos(2\pi /N)}.$$

This case is shown as a grey dashed line in figure 9*b*. By construction, it lies above both the *β* = 1 polar limit for *h* → 1 and the two-dimensional azimuthal limit.

We note that for large circles, the polar limit condition for pullers touching the wall in equation (4.15) is stronger than the azimuthal two-dimensional condition from equation (4.9), making the latter impossible to reach. In that case, the overall stability limit, as shown by the points, collapses on the polar stability limit, denoted by the dotted lines: this is exactly the case for *N* ≥ 7. By contrast, for five or six pullers close to the wall, the breaking of the original circles occurs through a combination of the two instabilities and we see in figure 9 that the points showing the numerical stability limit are higher than the lines for both azimuthal or polar stability. In this case, the coupling of the otherwise stable polar and azimuthal modes leads to the instability.

#### 4.5.2. Pushers

**Figure 10.**
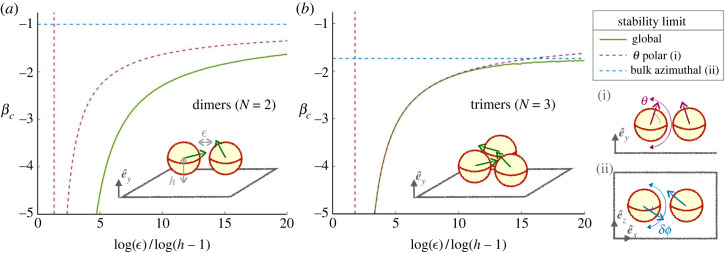
Stability of dimers and trimers of pushers above a wall. Critical squirmer parameters *β*_*c*_ for stability (*a*) dimers (*N* = 2) and (*b*) trimers (*N* = 3) as a function of the ratio ln⁡ϵ/ln⁡(h−1), with ϵ the spacing between the squirmers and *h* the hovering height. The purple dashed line represents *θ*-stability from equation (4.14), as in sketch (i). Above this line, the equilibrium angle *θ* exceeds *π*/2, leading to a polar break-up. The blue dashed line shows the limit for two-dimensional azimuthal stability from equation (4.9), shown in sketch (ii). The global stability condition is plotted in a continuous green line. For trimers, it lies at the minimum of the two previous conditions. For dimers however, it is significantly below both dashed lines and is then set by the azimuthal stability of the system when taking into account the value of the tilt angle *θ* set by the presence of the wall, as in equation (4.13).

Moving now to the case of pushers, we obtain numerically that, when too close to a wall, pushers reorient towards increased values of *θ* and eventually cross the threshold for stability given in equation (4.13). They therefore lose their stability when approaching a wall, as shown in figure 10. The wall now plays a destabilizing effect on dimers (*N* = 2), figure 10*a*, and trimers (*N* = 3), figure 10*b*. The conditions for stability are again given by equation (4.14) for finding an inclination θ*, which has to be below *π*/2 for polar stability, and equation (4.13) for the circle of tilted pushers at θ* to be stable against fluctuations of *δϕ*. The effect of the wall is particularly strong for dimers: when subject to lubrication interactions with a wall, the dimer can only be stable for *β* < −5/3, as opposed to *β* < −1 in two dimensions. Dimers above a wall destabilize through a combination of the polar *θ*-instability, which tilts the swimmers, and the azimuthal instability, which turns them outwards. Furthermore, dimers can only be stable when the squirmers are closer together than to the wall, namely, for $h>\epsilon^{4/3}$. For trimers, the two-dimensional stability limit can be reached for small ϵ, but all trimers are unstable if $h<\epsilon^{16/9}$. Shen & Lintuvuori [48] observe dimers and trimers of pushers above a boundary, with the trimers forming closer to the boundary, which is consistent with our results. However, the swimmers are not oriented radially in these cases, so any further comparison would require a dynamic analysis.

### 4.6. Strong confinement stabilizes circle clusters of pullers

We have shown that sedimentation can stabilize circles of pullers as a result of hydrodynamic reorientation from the bottom wall, but that it destabilizes otherwise stable dimers and trimers of pushers. Another common situation in which microswimmers encounter solid walls is that of confinement. To account for this, we now study the stability of circles of squirmers enclosed between two parallel walls. An advantage of this configuration is that the separation between the walls and the squirmers can be easily controlled experimentally, as opposed to the floating height of sedimented squirmers which can only be set through a careful calibration of the density of the swimmers. In addition, the distance to confining wall is less sensitive to the tilt angle *θ* of the squirmers.

We first note that pushers are destabilized when confined as is the case in the presence of a single wall. They again tilt along the polar direction and become sensitive to azimuthal perturbations. In particular, circle clusters of pushers in strong confinement become unstable regardless of swimmer strength and number.

**Figure 11.**
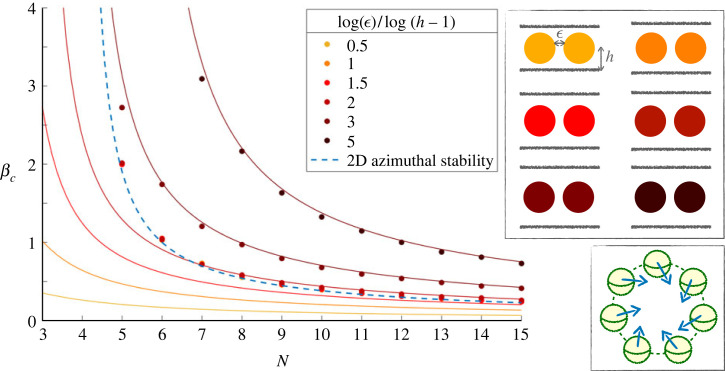
Stability of circle of pullers under strong confinement. Critical squirmer parameter *β*_*c*_ for stability as a function of the spacing between the squirmers ϵ and hovering heights *h*. For each ratio ln⁡ϵ /ln⁡(h−1), the symbols show the value of *β* above which the circle is stable, the dotted lines show the critical *β* value for theoretical stability in *θ* of equation (4.16). Example configurations with ϵ=0.5 are shown in the top inset. The blue dashed line represents the limit for the two-dimensional azimuthal stability from the previous section (equation (4.9)), with the corresponding reorientation sketched on the right.

We, therefore, turn to the clusters of pullers, and run simulations with different confinement strengths; the configurations and results for stability are shown in figure 11. Depending on the confinement, size of the cluster and squirmer strength, we observe that either the polar or the azimuthal instabilities can lead to cluster break-up, the former by increasing |*θ*| above *π*/2 and the latter by bringing *δϕ* beyond *π*/2 − *π*/*N*. Broadly, less confined systems are destabilized by the polar instability, as in the bulk, but as *β* and *N* increase, the azimuthal instability takes over. Finally, large confined clusters of strong pullers appear to be stable in our simulations, hinting once again at a stabilization of clusters of pullers in the presence of boundaries. More precisely, reducing either *β* and *N* favours the azimuthal instability with respect to the azimuthal one.

To rationalize these results, we first focus on the polar mode. We recall that, in the presence of a single wall, squirmers in circles of six or more reorient together towards increased values of *θ*. Now with two walls, we instead find that the tilting of neighbouring squirmers alternates between +*δθ* and −*δθ*. This makes intuitive sense as the system is now symmetric with respect to *θ* = 0, which is therefore always a fixed point. Our simulations show that if the values of *h* or *β* are too low, the clusters are unstable because of this alternating polar tilt, while they become stable for large *β* and strong confinement. This observation can be quantified theoretically by summing the torques from equations (4.1), (4.2), (4.4), assuming *δϕ*_*i*_ = 0, to find the stability limit

4.16 $$\beta >\frac{5L\sin(\pi /N)}{8-5L\sin^{2}(\pi /N)},$$

with L=ln⁡ϵ/ln⁡(h−1). We plot this in figure 11 as coloured lines for each wall separation. We see that our theoretical result does predict the numerical limit for stability for sufficiently high squirmer parameters and cluster sizes.

However, to account for very strong confinement, in particular for smaller clusters of weaker swimmers, our simulations show that we have to turn to the azimuthal instability. We then incorporate the two-dimensional azimuthal instability as given by equation (4.9) as a dashed line in figure 11. The two-dimensional condition predicts very well the destabilization at very strong confinement for h≤ϵ.

### 4.7. Some compact cluster configurations

**Figure 12.**
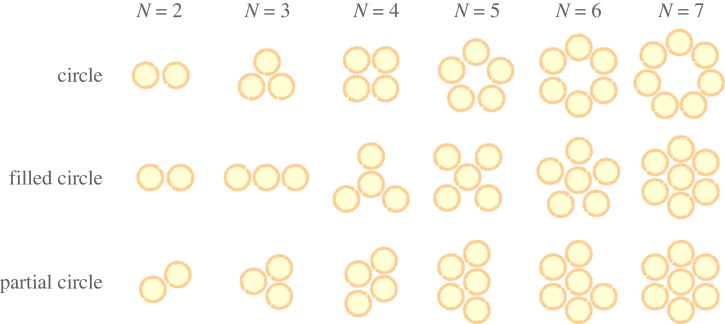
Cluster configurations investigated in our simulations, for up to seven squirmers. In the first one, the spheres lie on a circle, while the second includes a central squirmer. Finally, the partial circle state, which is observed experimentally, is a hexagonal structure that fills up with increasing *N*.

While the circle is a natural system to study, clustering squirmers can adopt other configurations. In two dimensions, the circle is the configuration that is symmetric for all *N*, but in three dimensions, we can also consider a filled circle, where an additional vertical squirmer is located at the centre. In experiments on the other hand, droplets are observed to adopt compact, rather than symmetric, configurations [40]. Typically, these take the form of ‘partial circles’, where the external spots of the filled hexagon fill in progressively. The circle, filled circle and partial circle configurations are depicted in figure 12 for up to seven squirmers. We can extend our simulations to these two additional systems, setting the minimal distance between two squirmers to ϵ. We ensure that hydrodynamic reorientation interactions occur between any neighbouring squirmers separated by less than a radius. Our default initial configuration is symmetric for the filled case, with the central squirmer facing upward or downward. For the partial circle, we make all the squirmers initially point to the barycentre of the cluster.

Importantly, our simulations reveal that no configuration, confined or not, can be made stable by the adjunction of a central squirmer, or by the reorganization into a compact structure. In particular, all puller clusters are still unstable in the bulk, and small clusters *N* ≤ 4 are unstable regardless of confinement. We also find that both the filled and partial circle clusters are unstable for *N* ≤ 7, regardless of confinement. Larger filled circles close to one or two walls can be stable according to our definition, but the inside squirmer will not stay upright: its orientation will be set by the boundaries, either tilted above one wall or horizontal in confinement. That system is, expectedly, less stable than the circle. If a stable final state exists, for sufficient confinement, *β* and *N* ≥ 8, the external squirmers point inward, and the inside one outward: some rearrangements might occur, and the group would be translating.

For pushers, the stable configurations are that with two or three squirmers only, including the unlikely full circle for *N* = 3, where three squirmers are aligned. We also find that the presence of a central squirmer tends to increase the growth rate of the azimuthal instability.

We therefore obtain that the circle stability generally acts as a lower bound for the stability of more compact configurations. Only local configuration changes in position instead of orientation could lead to less regular cluster shapes, and, possibly, further stabilize groups of squirmers. The stable regions and instabilities for these circular configurations are summarized in table 1.

## 5. Conclusion

Past studies have addressed the gravity-induced dynamics of a single squirmer above a wall. We revisit the equilibrium states for this system, defined as a pair of height and orientation, to be used later as initial conditions for the analysis of squirmer pairs. Including vorticity and velocity contributions from hydrodynamic interaction with the wall, as well as gravity and self-propulsion, yields equations for the equilibrium states. We find three possible equilibria: tilted swimming, upright floating, and sinking. In the first two, which occur for moderate gravity strength relevant to experimental systems, the squirmers are away from the wall and can thus be described in the far field. A range of weak pushers remain inclined with respect to the vertical and swim above the boundary. Decreasing the squirmer strength leads to a decrease in the tilting, reaching an upward floating state for weaker pusher, neutral and puller swimmers. By contrast, as squirmers sediment and reach low swimming heights, they must be studied in the lubrication limit, which is reached for both higher gravity and squirmer strength. The swimmer is then close to the wall (*h* < 2*R*) in an upright position for weak pushers and pullers, or tilted in the case of strong pullers.

The focus of this paper is the interaction of multiple squirmers above a boundary: a natural system consists of two squirmers in a symmetric position, starting from their far-field equilibria. Their orientation and speed are initially set by gravity, self-propulsion, and interaction with the wall, and they are modified by their hydrodynamic coupling. In the far field, we represent squirmers as hydrodynamic singularities—a force dipole and a source dipole—with their image on the boundary used to enforce the no-slip boundary condition. We first study the orientation and height of the pair at equilibrium when the distance between them is prescribed. We find that hydrodynamic interactions, whether with other swimmers or boundaries, primarily affect squirmers through reorientation rather than added velocity contribution. This is intuitive since velocity is dominated by self-propulsion and its competition with gravity, whereas a bulk squirmer has no intrinsic orientation and therefore torques are set by hydrodynamic interactions. Our model predicts that swimmers that were tilted on their own now reorient upwards when interacting as pairs. By contrast, pullers that float upwards on their own, tilt towards their neighbours as the separation between them decreases.

We then run planar dynamic simulations in which the motion of a symmetric pair of weak squirmers interacting in the far field is constrained to a vertical plane. While the time-dependent swimmer–swimmer interactions reflect the steady pair equilibrium at a fixed distance, we find that initial conditions play a critical role here. Indeed, initially tilted pushers already swim toward each other, with self-propulsion faster than the reorientation. They then collide before having significantly reoriented. Most of their interaction occurs in the contact (short-range) regime, leading to an upward reorientation and a subsequent lift-off, as expected from the equilibrium state. On the other hand, upright swimmers (weaker pushers, neutral squirmers and all pullers) repel one another above a critical distance *D*_*c*_, which increases for decreasing *β* (even reaching infinity for some pushers): if they start far apart, they turn away and separate. By contrast, below *D*_*c*_, the hydrodynamic interaction is attractive, and the swimmers tilt towards and approach each other. They then collide and keep sinking significantly after the contact. Because these swimmers had no horizontal velocity initially, the encounter is slower than that of tilted pushers.

The hydrodynamic far-field approach for a pair of squirmers, despite the obvious simplification from symmetry and two dimensions, yields rich dynamics for the explored range of squirmer parameters. It underlines the importance of initial conditions, and consequently of single-squirmer equilibria, in controlling multiple squirmers encounters. We also note the importance of considering not only discrete but continuous variations for *β*, in order to not overlook some features of the collective behaviour. In suspensions subject to gravity, collisions are generically expected: for tilted swimmers, they occur at random, because self-propulsion occurs faster than hydrodynamic reorientation while for upright ones they result from medium-range attractive interactions. Regardless of the squirmer’s strength, the majority of the interaction and reorientation occurs after the collision. Such short-range dynamics are better modelled in the near-field: we therefore investigate lubrication interactions between nearby swimmers (in the presence of boundaries or not).

Once the likeliness of collisions has been established, we study whether they are transient or long-lived, namely, if close-by squirmers form hydrodynamic clusters or instead break away. We consider a higher number of swimmers positioned in a circle. Since our two-swimmer analysis revealed that hydrodynamic interactions induce primarily viscous torques, as opposed to forces, we focus on the three-dimensional swimmer reorientations, keeping their positions fixed on the circle.

A cluster of swimmers is deemed stable if, when initially pointing radially inward, their orientation does not flip outside the circle. Two different modes can make the system unstable. In the azimuthal one, vertical tilting reaches and exceeds the upward orientation, while in the polar one, a large reorientation of at least one swimmer occurs in the plane of the circle. We study the system numerically, varying the number of squirmers *N*, their strength *β* and their separation ϵ. We also consider the effect of adding one boundary in the near field, as well as of confinement by two walls. In all cases, we complement the dynamic simulations with linear stability analysis and derive theoretically the boundaries for the onset of both polar and azimuthal instabilities.

Again, the dynamics are strikingly different for pushers and pullers. In the bulk, only dimers and trimers of pushers can be stable, whereas pusher larger groups break through a polar reorientation and circles of pullers all destabilize through azimuthal instability. Adding a boundary in the near-field of the pullers circle sets the vertical orientation and can prevent the bulk azimuthal instability. Since pullers’ circles of five squirmers (and more) are stable to polar instability for sufficiently large values of *β*, the boundary enables the formation of large stable clusters of pullers. Stability can be enhanced by incorporating a second wall. Under sufficiently strong confinement, the limiting instability for the puller circles is then no longer azimuthal, but polar, as in a two-dimensional system. By contrast, walls always destabilize dimers and trimers of pushers through a polar instability. Confined pairs and trios of pushers can be stable only for higher squirmer strength and small swimmer–swimmer separation. We summarize in table 1 all these results for the orientation stability of squirmers circles in two dimensions, in the bulk, above one wall and in confined configurations.

We also extend our simulations to more compact shapes of clusters that can be observed experimentally, including with a central squirmer. Such compact structures are all more unstable than the corresponding circle configurations.

We expect these results to supplement results from large-scale numerical simulations, and help rationalize results in the collective dynamics of swimmers, including the stability of pusher dimers and trimers above a boundary and the instability of pullers observed by Kuhr *et al.* [41]. Similarly, pushers in quasi-two-dimensional confinement can be driven to cluster only through close packing [27]. For a more accurate comparison with these simulations, an extension to this model would quantify the effect of Brownian noise on the stability, with dimers and trimers of pushers expected to eventually break because of noise and collisions with additional squirmers.

Regarding experiments, colliding oil droplets in [38] are seen to reorient upwards and lift off the wall. This is in agreement with the far-field dynamics of weak pushers. Yet, when three or more swimmers collide, experiments show the progressive formation of stable circle clusters, which grow from a dimer or trimer nucleus. By contrast, we predict that such clusters would be unstable from a purely hydrodynamic perspective. The presence of a wall is necessary to observe droplet clusters. As the wall tends to reorient the weak pushers upright, the boundary could help mitigate the effect of the azimuthal hydrodynamic instability predicted here. However, the mechanism that holds the cluster together has still not been elucidated. This sets a clear challenge to the effectiveness of using a purely hydrodynamic squirmer model to study the interactions between artificial microswimmers that have complex microscopic characteristics, in particular at close range [59]. Extensions of existing models coupling hydrodynamic interactions to physico-chemical interactions [10,35,60] are therefore needed to fully unravel the collective behaviour of self-propelled swimmers.

## Data Availability

The Matlab code used for the figures and simulations of clusters is published in Zenodo, https://doi.org/10.5281/zenodo.7979564 [56]. The data are provided in electronic supplementary material [61].
